# Intraluminal negative pressure wound therapy and rectal anastomotic leak management: regular vac sponge and a nasogastric tube

**DOI:** 10.1308/rcsann.2024.0022

**Published:** 2024-05-01

**Authors:** F Soliman, T Zwiep

**Affiliations:** Department of Colorectal Surgery, London Health Sciences Centre, Ontario, Canada

## Background

Anastomotic leak following low anterior resection can be devastating for a patient, often requiring the need for anastomosis disconnection and end-colostomy formation. In selected cases, negative pressure wound therapy (NPWT) can be utilised to manage extraperitoneal-contained anastomotic leaks.^1^ However, commercially available intraluminal NPWT can be costly, with the cost per unit quoted at £250.20/Endo-SPONGE^®^ (B.Braun, Melsungen Germany),^1^ with average number of sponge changes between seven and ten. The cost is further compounded with use of reusable endoscopy equipment and disposables such as vac-canisters.^1^

## Technique

**Figure 1:**
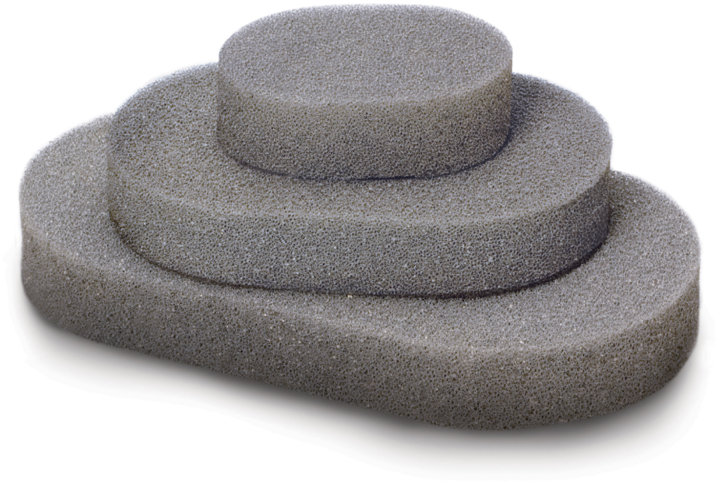
Granufoam™ sponge: cut into sections to create an appropriately sized intraluminal sponge to fix to the NG tube. A single medium-sized sponge can be used to complete ten sponge changes. NG = nasogastric

**Figure 2:**
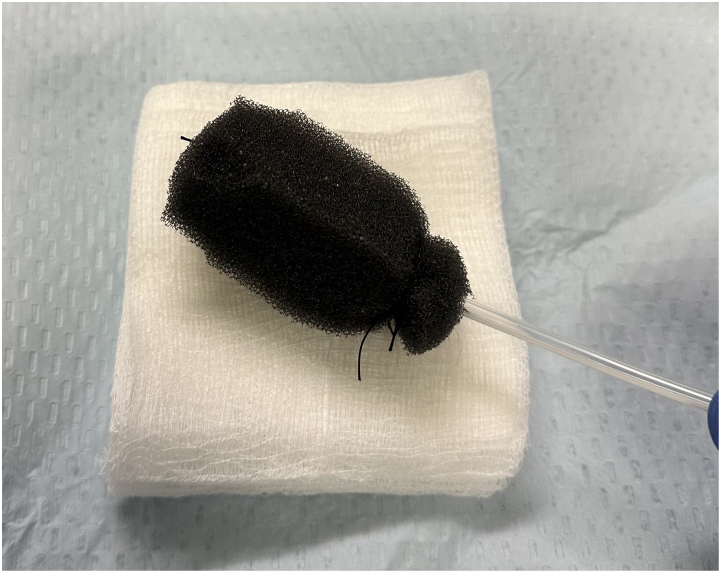
Intraluminal-NPWT sponge constructed with a 12ch nasogastric tube and secured at the top and bottom of the tube with 0-silk suture by transfixing through the NG tube. A channel down the middle is created using scissors, through which the NG tube can be threaded. The sponge is cut down and shaped to size according to the dimensions of the cavity to ensure normal mucosae is not subjected to NPWT. NG = nasogastric; NPWT = negative pressure wound therapy

A single medium Granufoam™ (3M, Maplewood, MN, USA), or equivalent, standard vac sponge dressing set (Figure 1) is used for the entire treatment therapy by cutting the required sponge size at each visit (total cost £17.38), together with ×10 12ch nasogastric (NG) tubes (total cost £19.99) and ×10 0-silk sutures (total cost £3) to construct and secure each NPWT (Figure 2). Total cost of disposables is £40.37 (+£46.96 canister cost) for a series of ten treatments, whereas total cost for ten treatments using the commercially available NPWT kit would be £2,522.90 (+£41.80 canister cost).

**Figure 3:**
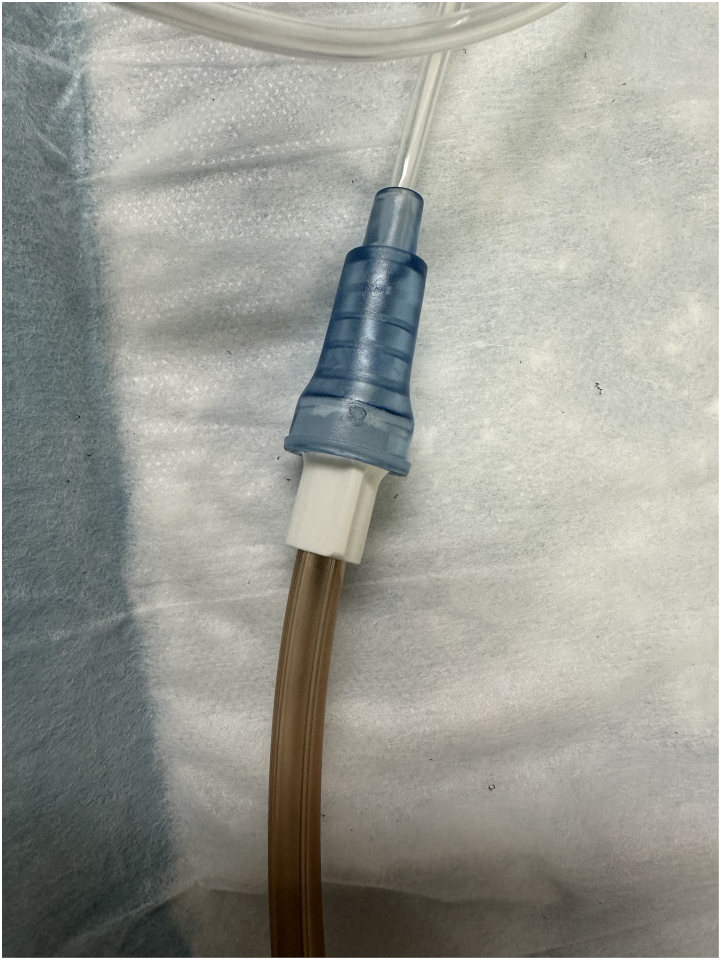
Vac canister attachment to NG tube. The vac canister comes with a specific locking attachment; however, the NG tube can be fitted over the top of the locking fitting to create an appropriate seal. NG = nasogastric

The NG tube is connected to a portable reusable vacuum unit (Figure 3) and set at a pressure of 100mmHg. Each sponge is changed every 48–72h until successful closure, often defined as a leakage cavity <1cm.

## Discussion

This is a cheaper and effective alternative to preprepared NPWT disposables, converting the contents of a single standard vac-sponge dressing pack to construct multiple cost-effective intraluminal NPWT devices. It is safe and feasible for colorectal anastomotic leaks.
